# Response characteristics of esophageal balloon catheters handmade using latex and nonlatex materials

**DOI:** 10.14814/phy2.12426

**Published:** 2015-06-15

**Authors:** Troy J Cross, Sophie Lalande, Robert E Hyatt, Bruce D Johnson

**Affiliations:** 1 Division of Cardiovascular Diseases, Mayo ClinicRochester, Minnesota; 2 Department of Kinesiology, University of ToledoToledo, Ohio; 3 Division of Pulmonary and Critical Care Medicine, Mayo ClinicRochester, Minnesota

**Keywords:** Balloon catheter, esophageal pressure, latex, nonlatex

## Abstract

The measurement of esophageal pressure allows for the calculation of several important and clinically useful parameters of respiratory mechanics. Esophageal pressure is often measured with balloon-tipped catheters. These catheters may be handmade from natural latex condoms and polyethylene tubing. Given the potential of natural latex to cause allergic reaction, it is important to determine whether esophageal catheter balloons can be fabricated, by hand, using *nonlatex* condoms as construction materials. To determine the static and dynamic response characteristics of esophageal balloon catheters handmade from *latex* and *nonlatex* materials, six esophageal catheter balloons were constructed from each of the following condom materials: natural latex, synthetic polyisoprene, and polyurethane (18 total). Static compliance and working volume range of each balloon catheter was obtained from their pressure-volume characteristics in water. The dynamic response of balloon catheters were measured via a pressure “step” test, from which a third-order underdamped transfer function was modeled. The dynamic ranges of balloon catheters were characterized by the frequencies corresponding to ±5% amplitude- and phase-distortion (*f*_A5%_ and *f*_*φ*5%_). Balloon catheters handmade from polyurethane condoms displayed the smallest working volume range and lowest static balloon compliance. Despite this lower compliance, *f*_A__5%_ and *f*_*φ*5%_ were remarkably similar between all balloon materials. Our findings suggest that polyisoprene condoms are an ideal *nonlatex* construction material to use when fabricating esophageal catheter balloons by hand.

## Introduction

The estimation of intrapleural pressure via its proxy, esophageal pressure (*Poes*) (Gillespie et al. 1973; Hurewitz et al. 1984; Hamid et al. 2005) allows for the determination of several indices of respiratory mechanics. These parameters include the resistance and compliance of the lungs and chest wall, intrinsic positive end-expiratory pressures, and respiratory muscle work and fatigue. These parameters offer insight into the energetics of respiratory muscle work in healthy and clinical populations (Johnson et al. 1992, 1993, 1996; Prigent et al. 2008; Cross et al. 2012, 2014) and have demonstrated clinical usefulness by helping guide mechanical ventilation therapy (Talmor et al. 2008; Plataki and Hubmayr 2011; Akoumianaki et al. 2014).

*Poes* is most often measured with an air-filled, balloon-tipped catheter positioned in the lower-third of the esophagus (Baydur et al. 1982). Esophageal balloon catheter systems are available for purchase from a number of medical supplies companies; however, due to low demand, these devices are relatively expensive (>$40 USD per catheter). Fortunately, the investigator may fabricate their own catheter balloons at a low cost via latex dip-molding (Mead et al. 1955; Lemen et al. 1974; Beardsmore et al. 1980) or by modifying a latex condom (Schilder et al. 1959) – the latter being the simplest approach. After the balloon is manufactured, it may then be affixed to the end of polyethylene tubing, completing the balloon catheter system. It is emphasized that the above methods describe only natural latex as a construction material. Given the allergenic potential of latex (Bykowsky 1996; Meeropol 1996; Binkley et al. 2003), it is important to establish whether esophageal catheter balloons can be fabricated in the laboratory, by hand, using *nonlatex* construction materials.

This short report examined whether esophageal balloon catheters handmade from *nonlatex* materials display similar static and dynamic response characteristics as those obtained from latex balloon catheter systems. In this study, catheter balloons were fabricated by modifying commercial condoms (Schilder et al. 1959) of the following materials: natural (latex), polyisoprene (synthetic latex), and polyurethane.

## Methods

### Fabrication of esophageal catheter balloons

Esophageal catheter balloons were constructed by modifying condoms using a method similar to that described by Schilder et al. (1959). In brief, the cuff and tip of the condom are cut away and discarded. The remaining sheath is then cut lengthwise to form a rectangular sheet. This rectangular sheet is folded over a talced glass rod (1.1 cm diameter) and is cemented together using a thin layer of adhesive (Pliobond®; Ashland Inc., Covington, KY). The cemented edge of the rubber sheet is trimmed to within ∽0.6 cm. The finished tube is then cut to a length of 12 cm. See Figure2 in Schilder et al. (1959) for an illustrated description of this procedure.

Catheter balloons were constructed from three different materials: (i) natural latex; (ii) synthetic polyisoprene; and (iii) polyurethane. Six catheter balloons were constructed for each of the above materials, yielding 18 total. The latex condoms were available in nonlubricated form (Trojan ENZ™; Church & Dwight Co., Princeton, NJ; nominal thickness = 0.07 mm). However, the polyisoprene (SKYN LifeStyles™; Ansell Healthcare, Iselin, NJ; nominal thickness = 0.07 mm) and polyurethane (Trojan™ Supra™ Bareskin™; Church & Dwight Co., Princeton, NJ; nominal thickness = 0.04 mm) condoms were only available in lubricated form. Lubricant was easily removed by washing the condoms in tepid water with dish soap (Dawn®; Proctor & Gamble Co., Cincinnati, OH) prior to balloon construction. Care was taken to avoid tearing the materials during washing.

### Assembly of the balloon catheter system

**Figure 1:**
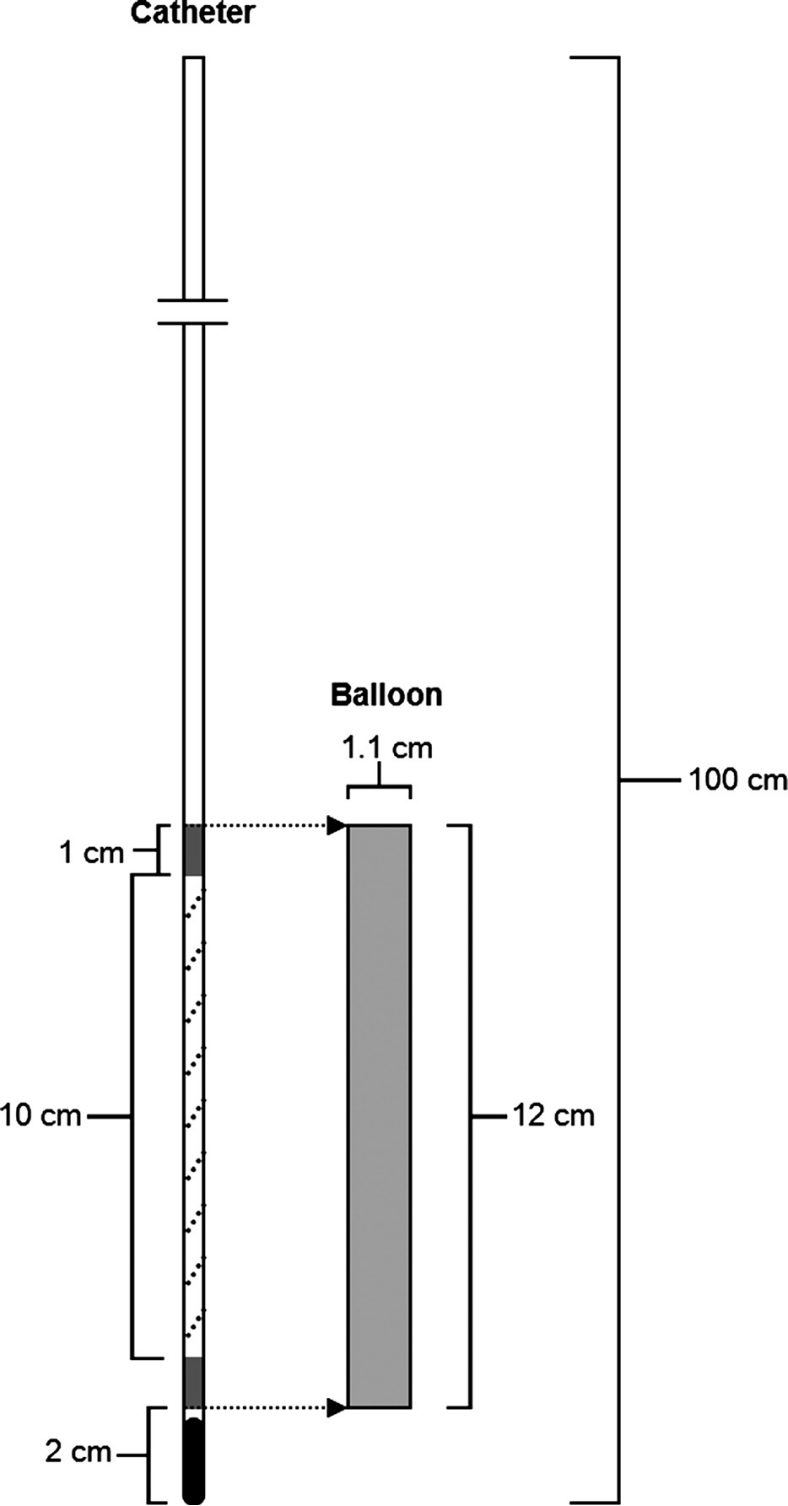
Simplified outline of the esophageal balloon catheter system.

A basic outline of the esophageal balloon catheter system is displayed in Figure1. Each catheter was prepared by cutting a 100 cm length of polyethylene tubing (PE 200, I.D. = 1.4 mm, Clay Adams Intramedic™; Becton, Dickson and Co., Franklin Lakes, NJ). At the distal end, a 2-cm-long section of the catheter surface was lightly scuffed using fine-grit sandpaper. A small amount of adhesive (Pliobond®; Ashland Inc.) was applied to this region to create a smooth rubber “nub” (solid-black portion, Fig.1). An airtight seal was ensured by allowing the cement to retract up the distal open-end of the catheter. Two 1-cm-long sections were marked, and lightly scuffed at sites 2 and 13 cm away from the distal end of the catheter tubing (dark gray portions, Fig.1). A very thin layer of cement adhesive was applied to the scuffed regions. These regions were used as attachment points for the esophageal catheter balloons. The section of catheter interposed between the two attachment points (10-cm long) was pierced repeatedly using a safety-pin in a spiraled manner. An esophageal balloon was then fed over the catheter, and positioned such that the balloon's ends were aligned with the prepared attachment points. Surgical suture wire was used to affix the balloon to these attachment points. A male luer stub was inserted into the proximal end of the catheter. The catheter's proximal end was directly interfaced with a pressure transducer for bench top testing. The completed system was tested for air leaks by submerging the distal end of the catheter in water, and inflating the balloon with air via a glass syringe at the proximal end. The balloon end of the catheter was positioned in a roughly horizontal orientation to minimize differences in transmural pressure acting on the balloon's surface due to the hydrostatic effect of water. Minor repairs were made by applying cement to the site of the air leak. If a leak was too large, or could not be repaired, the entire balloon catheter system was discarded. A catheter balloon system was deemed airtight if it could maintain a positive pressure of 5 cmH_2_O for a minimum of 10 min under water.

### Data acquisition

Intraballoon pressure was recorded by a differential pressure transducer (PX138-005D5V; Omega Engineering, Inc., Stamford, CT) connected to the proximal end of each balloon catheter system. The pressure transducer was calibrated using a water manometer before each test. The analog voltage output from the pressure transducer was sampled continuously at 4000 Hz (Powerlab 16SP, ADInstruments Inc., Castle Hill, Australia).

### Static response characteristics

**Figure 2:**
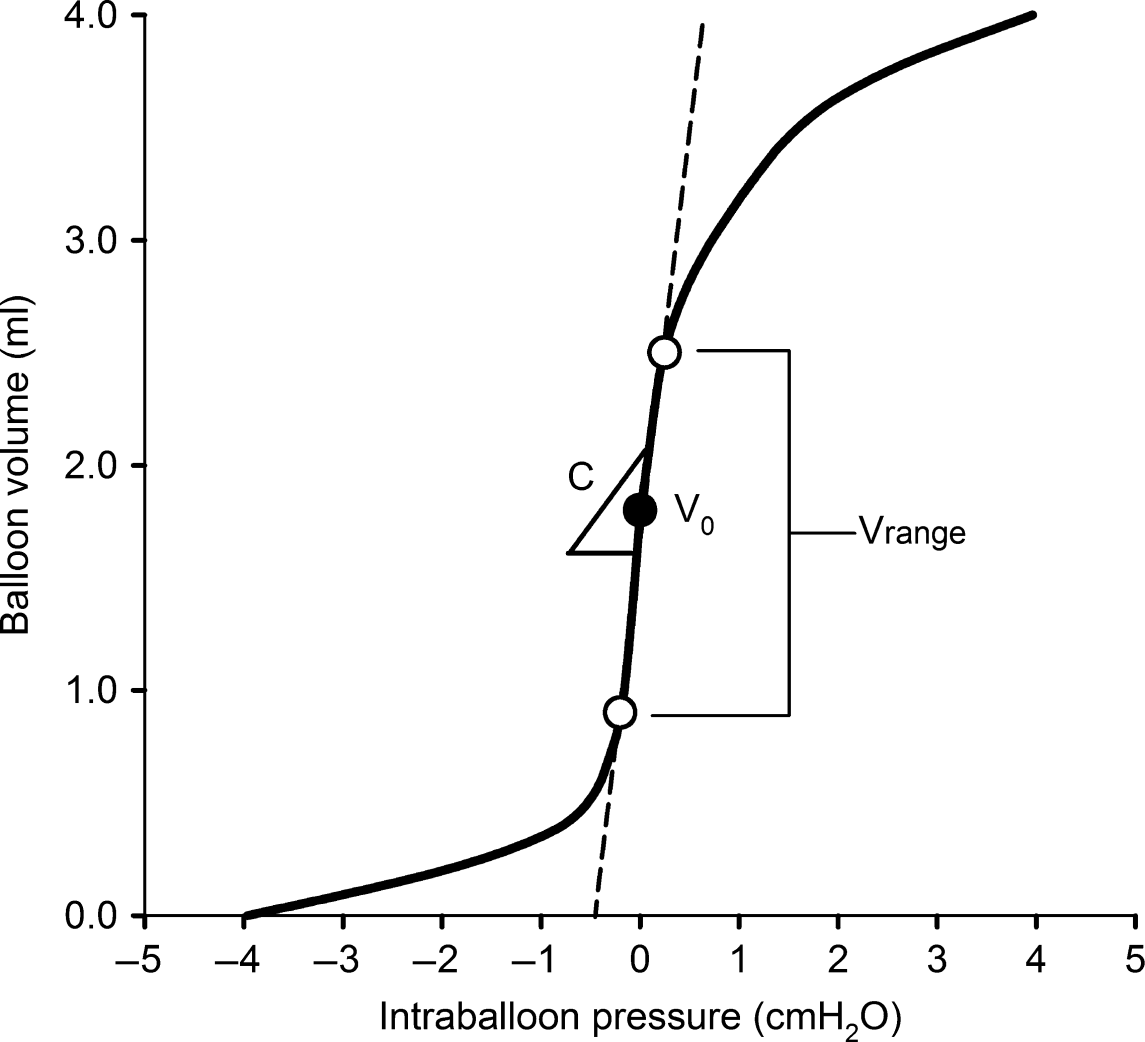
Static pressure–volume relationship of a representative esophageal balloon catheter handmade from a natural latex condom. The open circles represent the volumes corresponding to intraballoon pressures of ±0.2 cmH_2_O. *V*_0_, zero-pressure volume; *V*_range_, working range of balloon volumes; C, static compliance of balloon.

The static pressure–volume relationship of each esophageal balloon catheter system was assessed using previously described methods (Beardsmore et al. 1980). First, the distal end of the catheter was suspended in a glass jar filled with water, such that the balloon was completely submerged (horizontally) to a depth of 10 cm. At this depth, the balloon was considered empty of air. The distal end of the catheter was then removed from the jar and left to dry, so as to minimize surface tension acting on the balloon wall due to any residual water. While in air, the esophageal balloon was inflated via a glass syringe in 0.1–0.2 mL steps. Intraballoon pressure was recorded at each volume. The mean of five pressure–volume relationships were reported for each catheter system (Fig.2). The volume where intraballoon pressure first became atmospheric (i.e., ∽0 cmH_2_O) was defined as the zero-pressure volume (*V*_0_) (Beardsmore et al. 1980). The working range of the esophageal balloon (*V*_range_) was delimited by the volumes corresponding to intraballoon pressures of ±0.2 cmH_2_O. *V*_0_ typically occurred at the midway point of *V*_range_, indicating a relative symmetry of the pressure–volume curve. An index of static balloon compliance (*C*) was taken as *V*_range_/0.4 cmH_2_O.

### Dynamic response characteristics

**Figure 3:**
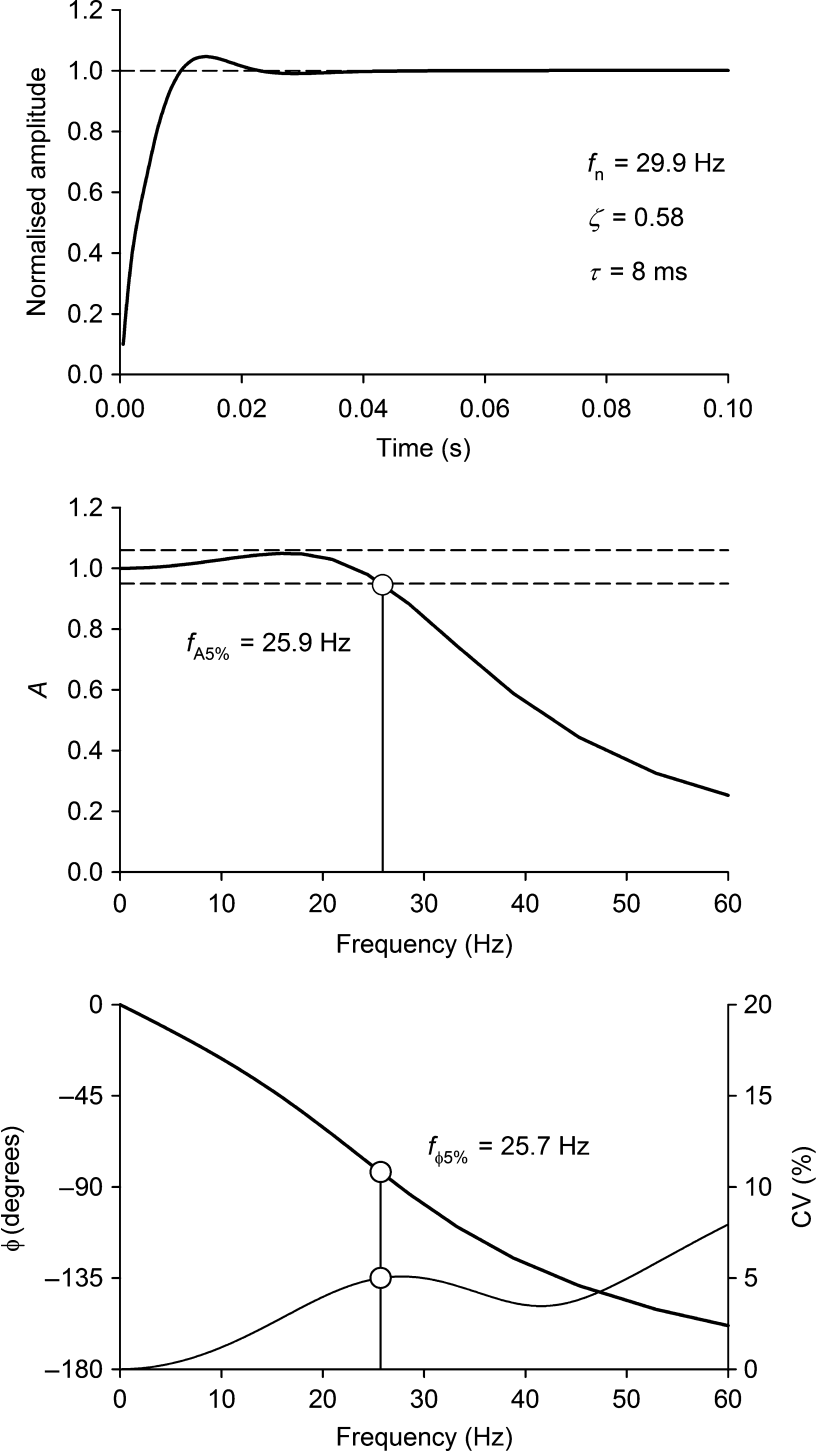
Step and frequency response curves of a representative esophageal balloon catheter handmade from a natural latex condom. The top panel displays the best-fit nonlinear function (Equation A2) used to describe the pressure “step” response of the esophageal balloon catheter. The middle and bottom panels illustrate the amplitude (*A*) and phase (*φ*) frequency response curves of the esophageal balloon catheter, as derived from Equation A3. *f*_n_, natural frequency; *ζ*, damping ratio; *τ*, time constant; *f*_A__5%_, ±5% amplitude error frequency; *f*_*φ*5%_, ±5% phase error frequency.

The frequency response characteristics of the balloon catheter systems were assessed via a pressure step test. The distal end of a balloon catheter system was suspended inside a rigid chamber, such that variations in chamber pressure would act directly on the surface of the catheter balloon, effecting changes in intraballoon pressure. A 3-cm hole was located on the bottom of the chamber. This opening was blocked by lodging a rubber stopcock into the hole. The balloon was inflated with a volume of air equal to V_0_. The chamber pressure was raised to ∽50 cmH_2_O, and intraballoon pressure was recorded for 10 sec. After which, the chamber was rapidly depressurized by knocking out the stopcock. Intraballoon pressure was recorded for a further 10 sec at this low chamber pressure (0 cmH_2_O). The pressure step test was repeated 6–10 times for each balloon catheter system. An ensemble-averaged step response was constructed from the repeated trials. The 10–90% rise-time (*t*10–90%) of each balloon catheter was obtained from the ensemble-averaged step response. Moreover, the step responses of all systems resembled that of a third-order underdamped linear system. These linear systems are characterized by a damping ratio (*ζ*), an *undamped natural frequency* (*ω*_n_), and a time constant of an additional first-order mode (*τ*). These parameters were obtained using the nonlinear regression fitting procedure described in the Appendix. Using this approach, we modeled the frequency response of each balloon catheter, from which we derived amplitude and phase error frequencies (*f*_A5%_ and *f*_*φ*5%_, respectively). These frequencies demarcate the upper-limit of the system's frequency response, beyond which there occurs significant (±5%) amplitude and phase distortion (see Appendix and Fig.3). Using *f*_A5%_ and *f*_*φ*5%_, the maximal working respiratory frequency (*f*_R,max_) was determined for each balloon catheter system (Equation A4). This value represents the highest respiratory frequency that each balloon catheter system will transmit the *Poes* waveform with minimal amplitude/phase distortion.

## Results

The coefficient of determination (*R*^2^) of the regression model fitted to step response data (Equation A2) was, on average, 0.99 (SD 0.01) for all balloon catheter systems. No appreciable difference in *R*^2^ was observed between balloon materials. The standard error of the estimate of the best-fit regressions was 0.58% (SD 0.01), 0.55% (SD 0.06), and 0.49% (SD 0.05) for latex, polyisoprene, and polyurethane balloon catheters, respectively. These results indicate that Equation A2 yielded a good fit to the measured pressure “step” responses, irrespective of the material used to construct the esophageal balloon. The static and dynamic response characteristics of the esophageal balloon catheter systems are reported in Table1. *V*_range_ and *C* were lowest for polyurethane esophageal balloon catheters. Conversely, *t*_10–90%_, *ζ, ω*_n_*, τ, f*_A5%_, *f*_*φ*5%_ and *f*_R,max_ were similar between all balloon catheter systems.

**Table 1:** Static and dynamic response characteristics of the handmade esophageal balloon catheter systems

	Material of the esophageal balloon catheter		
	Natural latex (*n* = 6)	Polyisoprene (*n* = 6)	Polyurethane (*n* = 6)
Static properties			
*V*_min_ (mL)	0.9 (0.6–1.4)	0.9 (0.6–1.0)	0.5 (0.4–0.8)
*V*_range_ (mL)	1.7 (1.4–2.4)	1.7 (1.4–2.0)	1.4 (0.8–2.2)
C (mL·cmH_2_O^−1^)	4.2 (3.5–6.0)	4.2 (3.5–5.0)	2.3 (1.6–3.0)
Dynamic properties			
*t*_10–90%_ (msec)	9.8 (9.5–10.2)	9.8 (9.3–10.3)	9.7 (9.5–10.0)
*f*_n_ (Hz)	30 (29–32)	30 (29–32)	30 (29–31)
*ζ*	0.59 (0.58–0.60)	0.59 (0.58–0.59)	0.59 (0.58–0.61)
*τ* (msec)	7.6 (7.2–8.0)	7.8 (7.1–8.3)	7.6 (6.5–8.2)
*f*_A5%_ (Hz)	26 (25–27)	26 (25–27)	26 (25–27)
*f*_*φ*5%_ (Hz)	25 (23–27)	24 (22–26)	24 (24–26)
*f*_R,max_ (breaths·min^−1^)	148 (138–160)	144 (134–154)	146 (141–155)

Values are presented as means with ranges in parentheses. *V*_min_, minimum volume where intraballoon pressure is −0.2 cmH_2_O; *V*_range_, working range of balloon volumes; C, static balloon compliance; *t*_10–90%_, 10–90% rise-time obtained from step response data; *f*_n_, natural frequency; *ζ*, damping ratio; *τ*, time constant; *f*_A5%_, ±5% amplitude error frequency; *f*_*φ*5%_, ±5% phase error frequency; *f*_R,max_, maximum respiratory frequency transduced by the catheter system with minimal amplitude/phase distortion.

## Discussion

This brief report demonstrates that the esophageal catheter balloons handmade (Schilder et al. 1959) from *nonlatex* condoms exhibit similar dynamic response characteristics to that observed for the latex balloon catheter systems. However, while dynamic performance was similar between systems, polyurethane balloon catheters displayed the lowest *C* and *V*_range_ of all three balloon materials under study.

### Static response

The static compliance (*C*) and, in turn, *V*_range_ of a balloon catheter is determined by the geometry of the balloon (i.e., length and radius), and the thickness and elastic modulus of the balloon material (Petit and Milic-Emili 1958; Beardsmore et al. 1980). Each balloon was manufactured to a similar length and radius in this study. Therefore, any differences in *V*_range_ and *C* were likely due to variations in wall thickness and elastic modulus between materials. Seeing that nominal wall thickness was comparable between natural latex and polyisoprene esophageal balloons (∽0.07 mm), it is not surprising that *C* and *V*_range_ were similar between these two materials. Furthermore, the molecule responsible for the favorable elastic properties of natural latex and polyisoprene condoms is the elastomer *cis*-1,4-polyisorpene – the primary constituent of both materials (Jacob et al. 1993). Polyurethane esophageal balloon catheters were the least compliant of all materials, evidencing the narrowest *V*_range_. Interestingly, the nominal wall thickness of these balloons was ∽0.04 mm, suggesting that the lower *C* of the polyurethane balloons was probably due to an inherently higher elastic modulus compared with the latex and polyisoprene condoms.

The gas inside of an air-filled balloon catheter will compress when exposed to a constant positive pressure (i.e., Boyle's law). Conversely, gas volume expansion occurs with the application of a constant negative pressure. There occurs a point where applied pressures are so extreme that the ensuing gas expansion/compression displaces the balloon volume outside of its working range (*V*_range_), ultimately causing artifact when measuring *Poes*. The maximum range of applied pressures may be estimated by: *P*_range_ = *V*_range_·(2·*α*)^−1^, where *α* is the volume-displacement coefficient of the total balloon-catheter-transducer system (∽0.003 mL·cmH_2_O^−1^ for each system). Accordingly, the estimated *P*_range_ for latex, polyisoprene, and polyurethane balloon catheters was ±278 cmH_2_O (range: 233–400), ±278 cmH_2_O (range: 233–333), and ±233 cmH_2_O (range: 133–367), respectively. Given that *Poes* rarely exceeds ±200 cmH_2_O during maximal static respiratory efforts (Agostoni and Rahn 1960; McCool et al. 1992; Nava et al. 1993; Brown et al. 2014), the *P*_range_ of handmade latex and polyisoprene esophageal balloon catheters seems adequate. On the other hand, we expect that our polyurethane balloon catheters would be inaccurate during extreme excursions in applied pressures (±150 cmH_2_O). One may easily widen the *V*_range_ (and thus *P*_range_) by slightly increasing the length and/or radii of polyurethane esophageal balloons during fabrication. However, the desire to increase the length/radii of an esophageal balloon must be tempered by the fact that too large a balloon volume can distend the esophageal wall. The additional transmural pressure offered by a distended esophagus would, in turn, overestimate *Poes* relative to intrapleural pressure (Milic-Emili et al. 1964).

### Dynamic response

Although it is important that an esophageal balloon catheter display ideal *static* elastic properties (e.g., high *C* and wide *P*_range_), the ideal system should also faithfully transmit *dynamic* changes in pressure without distorting the amplitude or phase of the original waveform. That is, the frequency response of an esophageal balloon catheter must be relatively uniform across the range of important frequencies. On this point, it is generally recommended that esophageal balloon catheters display a “flat” frequency response up to at least 15 Hz (Hamid et al. 2005). By modeling the esophageal balloon catheters as 3rd order underdamped linear systems (Equation A3), we were able to characterize the range of input frequencies wherein each balloon catheter system displayed minimal (±5%) amplitude and phase distortion. These frequencies, *f*_A5%_ and *f*_*φ*5%_, were remarkably similar between esophageal balloon catheters, irrespective of the condom material used to fabricate the balloon. From these data, we infer that our esophageal balloon catheters displayed tolerable amplitude- and phase-frequency responses up to roughly 24 Hz. These data, though informative, yield little practical insight into the practical ability of these balloon catheters to measure *Poes* in humans in vivo. We therefore calculated the maximal working range of respiratory frequencies which may be transduced by our catheter balloon systems with minimal amplitude/phase distortion (see *f*_R,max_ in Table1). Based on these results, it appears that our esophageal balloon catheters are able to faithfully reproduce *Poes* waveforms up to a respiratory frequency of roughly 140 breaths·min^−1^. Therefore, the dynamic response characteristics of our handmade balloon catheter systems appears suitable for measuring *Poes* in humans at rest, and during instances of overt tachypnea, such as that observed during maximal exercise or respiratory distress.

### Methodological considerations

It may be argued that the frequency response data presented in Table1 were limited by the rate at which the rigid chamber could be depressurized. This potential limitation was obviated by simultaneous measurement of chamber pressure during depressurizations. By using Equations A2 and A3, we observed that *t*_10–90%_, *ζ,* and *ω*_n_ of the rigid chamber was 2.8 msec, 0.526 and 608 Hz, respectively. These results indicate that the rigid chamber was depressurized at a rate faster than that observed in each balloon catheter system. We are therefore confident that the derived frequency responses of the esophageal balloon catheters (Table1) were not limited by the chamber's own frequency response function. It is also worth mentioning that the “airtightness” of our balloon catheters were assessed for a minimum of 10 min. However, in practice, one may wish to measure *Poes* over longer periods of time (e.g., hours or days). Thus, further studies are needed to confirm the airtightness of our handmade esophageal balloon catheters over longer durations than reported here.

### Recommendations

It is clear from the above that (synthetic) polyisoprene condoms are the ideal *nonlatex* materials to use when constructing esophageal balloons by hand (Schilder et al. 1959). Not only do these catheter balloons display comparable static elastic properties to those handmade with natural latex condoms, but also their amplitude- and phase-frequency responses are tolerable up to roughly 24 Hz. However, we must not discount the potential advantages of using a polyurethane condom as a *nonlatex* balloon material. As noted above, a practical solution to improving *V*_range_ and *P*_range_ of polyurethane esophageal balloons is to increase their length and radii during fabrication. These slightly larger balloons would offer several advantages over catheter balloons made from polyisoprene (and natural latex). For example, polyurethane condoms are nonporous and exhibit higher chemical resistance than either natural latex or synthetic polyisoprene condoms. The latter point is particularly important if the esophageal balloon catheter is exposed to the gastric contents for long durations (e.g., when measuring gastric pressures). Future investigations should examine the relative durability of polyisoprene and polyurethane esophageal balloons when exposed to low-pH solutions, such that their suitability for monitoring gastric pressures is assessed.

The construction of each balloon catheter required several hours of labor to accommodate for adhesive drying times, preparation of tubing sections, etc. Importantly, the investigator may be efficient with their time by fabricating any number of catheters in parallel. Given that esophageal balloon catheters are technically *noninvasive* devices (i.e., they do not penetrate the endothelium), they may be appropriately disinfected by medical-grade antimicrobial solutions. As such, these catheters are amenable to repeated-use provided investigators take the necessary steps to disinfect each balloon catheter between uses.

## Conclusions

Given the potential for natural latex to cause allergic reaction (Bykowsky 1996; Meeropol 1996; Binkley et al. 2003), we determined whether esophageal balloon catheters could be handmade from *nonlatex* materials. We show here that esophageal balloon catheters handmade (Schilder et al. 1959) from (synthetic) polyisoprene condoms display static and dynamic properties similar to those manufactured using natural latex condoms. Thus, we propose that (synthetic) polyisoprene condoms are the ideal *nonlatex* materials to use when constructing esophageal balloons by hand.

## Appendix

### Calculating the Dynamic Response of Balloon Catheters

The step response of all catheter systems was characterized by an transient undershoot in intraballoon pressure, followed by a brief period of “ringing” before settling at a final value of 0 cmH_2_O. This observation suggested that the behavior of the balloon catheter systems was similar to that of an underdamped second-order linear system. Second-order linear systems are characterized by their damping ratio (*ζ*) and the *undamped natural frequency* (*ω*_n_). These two quantities may be estimated from the step response via least-squares fitting of the following equation:

A1

where *P* is intraballoon pressure and *TD* is the absolute time (s) at which the step change in pressure occurred. Least-squares fitting of the above nonlinear equation was performed using commercial software (OriginPro 8.5; OriginLab Corp., Northampton, MA). Before the nonlinear fitting procedure, step response data were inverted and normalized to represent a positive step change of unit magnitude. Data occurring before intraballoon pressure had changed by more than 15% of the full-scale response were removed from further analyses. These data were removed because an instantaneous pressure “step” could not be guaranteed during the initial moments of depressurizing the chamber. Preliminary analyses revealed that Equation A1 did not provide a satisfactory fit to the observed step responses. Instead, the Equation A1 was expanded to incorporate a first-order mode, such that:

A2

where *τ* is the time constant of the additional first-order mode. The best-fit parameters *ζ, ω*_n_, and *τ* were used to obtain the frequency response of the balloon catheter system by using the third-order transfer function:

A3

where *G* is a complex number, and *s* is the Laplace operator and may be replaced by angular frequency (rad·sec^−1^). Bode amplitude (*A*) and phase (*φ*) plots were constructed by taking the absolute and complex arguments of *G*, respectively. Bode plots were evaluated over the frequency interval of 0–100 Hz.

We identified discrete frequencies at which *A* and *φ* fell outside tolerable error margins (Fig.3). The amplitude error frequency (*f*_A5%_) was defined as the first frequency where *A* fell outside the interval of 0.95 to 1.05 (Fry 1960; Hipkins et al. 1989; Hartford et al. 1997): that is, the bandwidth of frequencies where the amplitude-ratio may be considered “flat” to within ±5% (Fig.3 middle panel). The phase error frequency was obtained by examining the phase distortion introduced by the balloon catheter system. Phase distortion may be observed when *φ* deviates from a straight line of negative slope. We used an iterative regressive approach to find the input frequency where *φ* no longer followed a negative phase slope. An initial linear regression model was fit to the first three *φ* values on the Bode phase-frequency plot (starting from 0 Hz). The coefficient of variation (CV) of the best-fit line was determined as the root mean square of residual scores divided by the mean of the corresponding *φ* values. This process was repeated by including proceeding *φ* and frequency points in the regression model one at a time. CV was calculated during each permutation. This procedure continued iteratively until CV exceeded a value of 5% (i.e., where a linear segment no longer explained *φ* data). The discrete frequency corresponding to this point was defined as the phase error frequency; that is, *f*_*φ*5%_ (Fig.3 bottom panel). To accurately reproduce a pressure waveform, it is a generally accepted that a catheter system display a “flat” frequency response up to the 10th harmonic of the fundamental frequency of the original waveform. The fundamental frequency of the *Poes* waveform would be the spontaneous respiratory frequency. Based on this rule-of-thumb, we calculated the maximal working respiratory frequency (*f*_R,max_) that can be measured by each esophageal balloon catheter as:

A4

where the min argument returns the lower of either *f*_A5%_ or *f*_*φ*5%_.
